# Splenic rupture and fungal endocarditis in a pediatric patient with invasive fusariosis after allogeneic hematopoietic stem cell transplantation for aplastic anemia: A case report

**DOI:** 10.3389/fped.2022.1060663

**Published:** 2022-12-02

**Authors:** Maurice Hannemann, Dunja Wilmes, Frank Dombrowski, Jürgen Löffler, Alexander Kaminski, Astrid Hummel, Lena Ulm, Jürgen Bohnert, Volker Rickerts, Jan Springer, Holger N. Lode, Karoline Ehlert

**Affiliations:** ^1^ Department of Pediatric Hematology and Oncology, University Medicine Greifswald, Greifswald, Germany; ^2^ Division for Mycotic and Parasitic Agents and Mycobacteria, Robert Koch Institute, Berlin, Germany; ^3^ Institute of Pathology, University Medicine Greifswald, Greifswald, Germany; ^4^ Department of Internal Medicine II, University Hospital Würzburg, Würzburg, Germany; ^5^ Department for Heart and Vascular Surgery, Klinikum Karlsburg, Karlsburg, Germany; ^6^ Department of Internal Medicine B, University Medicine Greifswald, Greifswald, Germany; ^7^ Institute of Microbiology, University Medicine Greifswald, Greifswald, Germany

**Keywords:** invasive mold infection, fusarium, endocarditis, splenic rupture, hematopoietic stem cell transplantation, case report

## Abstract

**Background:**

Invasive mold infections are a well-known and life-threatening condition after allogeneic hematopoietic stem cell transplantation (HSCT). While *Aspergillus* species are recognized as predominant pathogens, *Fusarium* species should also be considered due to their broad environmental distribution and the expected poor outcome of invasive fusariosis. Particularly, splenic rupture as a complication of disseminated disease has not been reported yet.

**Case presentation:**

Two weeks after allogeneic HSCT for severe aplastic anemia, a 16-year-old boy presented with painful, erythematous skin nodules affecting the entire integument. As disseminated mycosis was considered, treatment with liposomal amphotericin B and voriconazole (VCZ) was initiated. Invasive fusariosis was diagnosed after histological and previously unpublished polymerase chain reaction-based examination of skin biopsies. Microbiological tests revealed *Fusarium solani* species. Despite stable neutrophil engraftment and uninterrupted treatment with VCZ, he developed mold disease-associated splenic rupture with hypovolemic shock and fungal endocarditis. The latter induced a cardiac thrombus and subsequent embolic cerebral infarctions with unilateral hemiparesis. Following cardiac surgery, the patient did not regain consciousness because of diffuse cerebral ischemia, and he died on day +92 after HSCT.

**Conclusion:**

Invasive fusariosis in immunocompromised patients is a life-threatening condition. Despite antimycotic treatment adapted to antifungal susceptibility testing, the patient reported here developed uncommon manifestations such as splenic rupture and fungal endocarditis.

## Introduction

Bacterial, viral, and fungal infections are frequently observed in children and adolescents after allogeneic hematopoietic stem cell transplantation (HSCT). Invasive fungal disease is a life-threatening condition (1). Of particular concern are disseminated infections by mold species which may require the combination of drug treatment and surgical measures to achieve optimal outcomes (2). Major risk factors for infections by mold species are extended periods of neutropenia, the systemic use of steroids, and the presence of chronic graft-versus-host disease (GvHD) (1). While *Aspergillus* species represent the most predominant pathogens in these patients, *Fusarium* species also need to be considered due to their broad, environmental distribution (3). Here, we report a fatal course of *Fusarium solani* infection in an adolescent after allogeneic HSCT for aplastic anemia. A rupture of the spleen was observed in our patient as a hitherto unique manifestation of disseminated fusariosis. However, the most critical and finally fatal event in the post-transplant course was fungal endocarditis with subsequent embolization into the brain from a cardiac thrombus.

## Case presentation

A 16-year-old male patient was diagnosed with very severe aplastic anemia (VSAA) after a brief history of petechiae and pale skin. The results of the standard diagnostic procedures for acquired bone marrow failure had ruled out acute leukemia, Fanconi anemia, dyskeratosis congenita (DC), paroxysmal nocturnal hemoglobinuria (PNH), myelodysplastic syndrome (MDS), and a primary immunodeficiency (PID) syndrome. The patient's family history was uneventful. Allogeneic HSCT from an HLA-identical brother was postponed for several months as the family hoped that identifying a causal agent and its treatment could reverse the effects on the bone marrow. Supportive care during these few months included frequent, mostly weekly transfusions with platelets and packed red cells and antimicrobial prophylaxis with topical amphotericin B, oral fluconazole, and cotrimoxazole. The patient's neutrophil count was permanently below 500 µl. Consent for HSCT was obtained after 6 months of neutropenia when the patient had developed a threatening soft tissue infection by *Streptococcus anginosus* in his left hand, requiring surgical treatment.

The patient's allogeneic HSCT was performed with unmanipulated bone marrow (2.4 × 10^6^ CD34-positive cells/kg) from his HLA-identical sibling after conditioning with cyclophosphamide (CYC, 50 mg/kg/day for 4 days) and antithymocyte globulin (ATG, 15 mg/kg/day for 4 days). On day (day) −4, a first febrile episode in neutropenia occurred due to a bloodstream infection by *Pseudomonas aeruginosa*. The patient was successfully treated with piperacillin/tazobactam and tobramycin. Antimicrobial prophylaxis included aciclovir (post-transplant switched to foscarnet because of cytomegalovirus-positivity of donor and recipient) and micafungin (1 × 50 mg). For prophylaxis of GvHD, methotrexate (10 mg/m^2^/day on day +2, +4, +7) and ciclosporin A (CSA) were administered. On day +23, CSA was replaced by tacrolimus because of peripheral neuropathy. On day +8, the patient developed a second episode of fever with rising inflammatory markers. His blood cultures remained sterile. The patient's antibacterial treatment was switched to meropenem and teicoplanin resulting in rapid resolution of the fever. Two days later, a painful fissure between the 4th and 5th left toe appeared without any physical injury. Microbiological samples revealed the presence of multisensitive *Escherichia coli*, so the antibacterial treatment was maintained. The lesion became necrotic and cavernous with a maximum depth of 1.5 cm. On day +15, painful, erythematous skin nodules were found affecting the entire integument. As infectious metastases of fungal origin were suspected, micafungin was switched to liposomal amphotericin B (LAmB, 1 × 3 mg/kg, from day +31 5 mg/kg, stop date day +34) and intravenous (i.v.) voriconazole (VCZ, loading dose 2 × 6 mg/kg, maintenance therapy 2 × 4 mg/kg).

Several cutaneous biopsies were taken on day +17, which histologically proved hyalohyphomycosis, compatible with invasive aspergillosis. Serum Aspergillus-galactomannan antigen on day +21 was negative. One of the formalin-fixed paraffin-embedded (FFPE) skin biopsies was sent to the Mycologic Laboratory at the Robert Koch Institute Berlin for further analysis.

**Figure 1:**
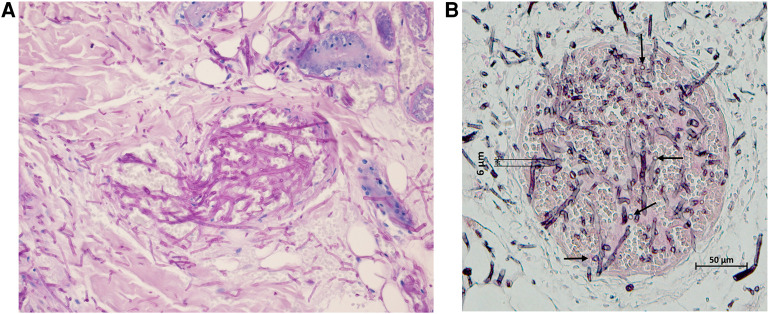
(**A**) Microscopic image of a skin lesion. Histologic examination of a skin biopsy following periodic acid–Schiff (PAS) staining shows the extravasation of hyphae from a blood vessel into the surrounding tissue. It is important to note that there is not any reaction of the cellular immune system against this fungal invasion. (**B**) Tissue biopsy of the cutaneous lesion showing vascular invasion and infarction by small, septated hyphae intermingled with chlamydoconidiae (indicated by the arrows) stained by Grocott's methenamine silver stain (magnification 10x), both day +17. Both images were obtained from the same FFPE (formalin-fixed paraffin embedded) tissue.

Histopathology after Grocott's methenamine silver (GMS) stain showed vascular invasion and infarction by strongly stained small, septated hyphae with acute angle branching and small, single-celled chlamydoconidiae (Figures 1A,B). DNA was extracted by the protocol described by Rickerts et al. (4) and studied by five different qPCRs. To document successful DNA extraction a qPCR detecting the human 18S rRNA gene was used (5). An internal amplification control DNA (IAC qPCR) was used to exclude PCR inhibition. Fungal DNA was amplified using a broad range 28S qPCR (primer 10F and 12R) (5–8), and the DNA was sent to a collaborating laboratory for two specific qPCR assays to detect *Aspergillus* DNA (7, 9, 10) and DNA of *Mucorales* (7, 11). No inhibition, defined as a delta CT of more than two cycles (5), was detected. The broad range qPCR amplified fungal DNA in duplicates in less than 40 cycles (CTs) with identical peaks in the melting curve analysis in the absence of positive no-template-, or extraction negative controls. The identification of the resulting sequences by BLAST search in GenBank (12) yielded an identity of 99.7% with the sequence of an ATCC *Fusarium solani* (accession number FJ345352) strain. Both the *Aspergillus* and *Mucorales* qPCRs were negative.

The patient's first VCZ level on day +21 6 days after the start of treatment was 12 mg/l. However, multiple further tests did not detect VCZ levels above 2 mg/L despite continuous intravenous administration of VCZ. To exclude false-negative results, VCZ tests were performed in two different laboratories revealing identical findings. After obtaining these low trough levels, VCZ was increased to 12 mg/kg i.v. until the patient's discharge to his home.

Neutrophil engraftment with full donor chimerism was documented on day +22. Massive capillary-leak syndrome with lung and peripheral edema and acute renal failure caused by the interaction of tacrolimus and i.v. VCZ required continuous venovenous hemodialysis from day +27 to +30 and invasive mechanical ventilation due to uremic encephalopathy. Despite these transient, however serious organ failures, the patient's antimycotic treatment was maintained without interruption with i.v. VCZ. Cultures of tracheal samples and wound swabs during this first period in the pediatric intensive care unit grew hyphomycetes, which were identified as *Fusarium* species. An antifungal susceptibility testing (AFST) was done and showed sensitivity to VCZ and resistance to LAmB.

**Figure 2:**
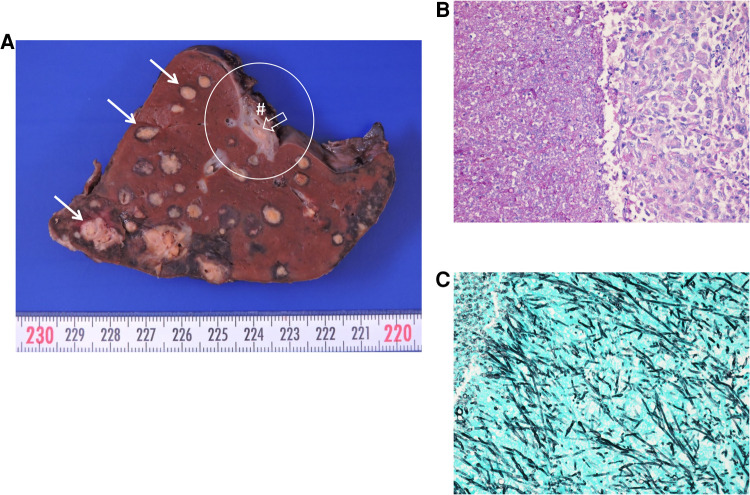
Macroscopic and microscopic image of the ruptured spleen. (**A**) Macroscopic image of the spleen. Solid arrows indicate multiple intravascular fungal lesions with perifocal bleeding. Asterisk shows the red pulp with dark brown discoloration consistent with transfusion associated hemosiderosis. Circle shows the splenic hilum with the lienal vein (hash sign), where fungal lesions outside the spleen can be identified (open arrow). (**B,C**) Microscopic images of a splenic lesion following periodic acid–Schiff (PAS). (**B**) and Grocott's silver staining (**C**) showing hyphae and chlamydoconidiae of *Fusarium solani*. The length of the lower border is 612 µm each.

Besides the cutaneous foci, ultrasound and computed tomography (CT) also showed mycotic lesions in his lungs, liver, both kidneys, and most impressively, in the patient's spleen. The magnetic resonance imaging (MRI) of his brain and an ophthalmological examination were unremarkable. On day +46, a sudden circulatory failure resulted in a life-threatening emergency caused by a splenic rupture as demonstrated in an abdominal CT scan. Most likely, the event was caused by an initial hemorrhage into one of the mycotic lesions of the spleen, followed by the rupture of the splenic capsule. A splenectomy was performed immediately. Diffuse involvement of the spleen with mold species was confirmed upon pathological examination (Figures 2A–C).

**Figure 3:**
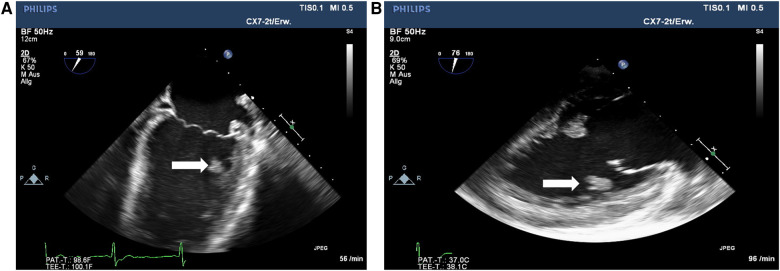
(**A** and **B**) Views on thrombus (white arrows) in the outflow tract of the left ventricle, day +80.

**Figure 4:**
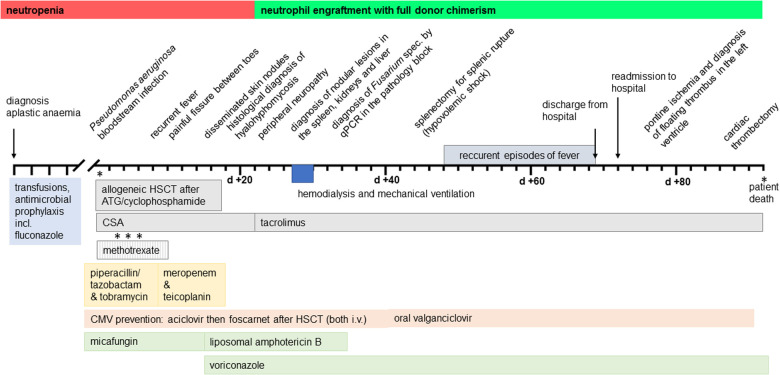
Synopsis of the clinical course and treatment. HSCT, hematopoietic stem cell transplantation; ATG, antithymocyte globulin; CSA, ciclosporin A; CMV, cytomegalovirus.

The patient's condition gradually improved; his skin lesions healed with residual small scars. However, several episodes of fever were noticed from day +48 until his discharge on day +69. Blood cultures and viral monitoring remained unremarkable. Imaging studies (MRI, CT, transthoracic echocardiography) did not reveal new findings. An episode of upper abdominal pain was caused by biliary tract obstruction, which was solved by an endoscopic procedure. The patient was discharged on day +69. He was re-admitted on day +72 because of reduced general condition and insufficient eating and drinking. Supportive treatment was initiated. On day +76, the patient experienced sudden right-sided hemiparesis caused by pontine ischemia. In transesophageal echocardiography (TEE), a floating thrombus in the outflow tract of the left ventricle with a size of 1.5 cm was found (Figures 3A,B). A multi-disciplinary case discussion was initiated. Considering the severe previous organ failures, a surgical procedure was not favored at first. The patient received low-molecular-weight heparin and broad empirical antibacterial treatment in addition to his antifungal therapy. He continued to have regular fever spikes; blood cultures remained sterile. In a follow-up TEE 7 days later, the size of the thrombus had increased. MRI revealed a second ischemic area in the cerebellum which was asymptomatic. The patient's situation was discussed with the patient, his parents, and each involved medical department. A cardiac surgical procedure with thrombectomy was regarded as unavoidable. Although the patient acknowledged the risk of the procedure, he clearly wished to take this option as he increasingly suffered from his condition. Despite broad analgesic treatment, including opioids, he complained about persistent, severe headaches and, in addition, felt miserable because of his unchanged hemiparesis. After the removal of the left ventricular thrombus on day +91 in a nearby cardiac surgery clinic, the patient did not regain consciousness. A CT scan revealed diffuse bilateral ischemia and edema in the brain. With respect to the expected poor neurological outcome, the parents decided not to proceed with a neurosurgical procedure. On day +92, the patient was disconnected from the respirator and died. The timeline of major events is provided in Figure 4.

A post-mortem examination was not permitted. Microbiological investigation of the thrombus confirmed the presence of *Fusarium solani* by culture. In a family conference a few months after the patient's death, the parents expressed their regrets about giving their consent to their son's allogeneic HSCT. They felt he should have been granted more time to enter the procedure in a more stable physical condition.

## Discussion

Despite continuous antimycotic, AFST-adapted treatment with VCZ, timely neutrophil engraftment after allogeneic HSCT, and two surgical procedures, this patient did not survive disseminated infection with *Fusarium solani*. It is possible that the acquisition of *Fusarium* happened through transcutaneous inoculation in the initial skin lesion between his toes. Unlike aspergillosis, cutaneous involvement has been described in around 85% of reported cases of *Fusarium* infection, particularly in immunosuppressed patients (13). Although the cutaneous lesions healed during treatment with VCZ and in the presence of neutrophils, the spleen remained most severely affected. This may be caused by the specific architecture of this organ. A splenic rupture due to *Fusarium* infection has not been described before. The patient's death was finally caused by embolic events in the brain originating from a thrombus in the outflow tract of the left ventricle with proven colonization by *Fusarium solani*.

*Fusarium* spores are widely distributed in soil, and air (3), but also in plumbing systems (14) and may cause infections in plants, animals, and humans (14–16). In most cases, the principal portal of entry are the airways, followed by skin and mucosal membranes (3). In HSCT recipients, the risk is highest in recipients of mismatched unrelated donor allogeneic transplants (17). Risk factors for invasive fusariosis in the early phase of allogeneic HSCT include the receipt of ATG, and in the late phase, nonmyeloablative conditioning regimen, GvHD, and previous invasive mold disease (2). Typically, the affected patients present with persistent or recurrent fever despite broad-range antibiotic therapy and the sudden appearance of multiple skin lesions, pneumonia, or sinusitis. Lung CT scan and cutaneous biopsies are indicated, and laboratory diagnosis relies mostly on the cultural isolation of the fungus from blood or skin lesions. Differential diagnosis with aspergillosis may be challenging, as both hyalohyphomycoses are histologically almost undistinguishable and serum galactomannan may be positive in both infections (18, 19). Current guidelines recommend treatment with VCZ (20). As the persistence of severe immunosuppression, particularly neutropenia, is the most important factor associated with the poor outcome of patients with invasive fusariosis, reduction or withdrawal of immunosuppression is recommended as early as possible.

In our patient, the diagnosis of infective endocarditis was maintained following the modified Duke criteria (21). Endocarditis caused by *Fusarium* species is rarely seen, and associated with a high mortality rate (22–25). Treatment includes long-term antifungal therapy and, if possible, surgery (26, 27). Few publications have reported this clinical manifestation before, the largest by Inano et al. in 2013 (22). In his retrospective analysis, five of seven affected patients did not survive, demonstrating the poor prognosis of this particular site of infection. Of the two surviving patients, one had a surgical resection, the second did not. In their paper, the authors suggest the combination of VCZ with terbinafine (TBF) based on in-vitro-studies and their experience in an own patient. Randomized clinical trials to answer this question are not yet available. In our patient, it remained unclear when exactly the endocarditis developed and whether the splenic rupture contributed to the dissemination of *Fusarium* species into other organs.

As *Fusarium* is intrinsically resistant to a broad range of antifungals, and the optimal treatment strategy remains a major challenge, AFST may be a helpful tool to guide the treatment (16, 28). The global guidelines recommend as first-line treatment VCZ with therapeutic drug monitoring (TDM) or LAmB (28). When using VCZ it must be considered that low or even unmeasurable plasma levels can often be found. This is partly due to ultrafast metabolizers (29), but essentially this phenomenon is not completely understood. Some retrospective studies have identified a relationship between VCZ trough concentrations and clinical outcome (30–32), some others have not (33–35). Therefore, treatment of these often critically sick patients with disseminated mold disease may be complicated by the lack of reliable data. Different combination therapies, as VCZ plus TBF (22), CAS plus amphotericin B deoxycholate (dAmB) (36), dAmB plus VCZ (37–39) or dAmB plus TBF (40) have been described in limited case reports. More recent data justify the initial combination of VCZ with TBF (22), CAS, micafungin, or posaconazole, particularly until the targeted range of VCZ trough concentration is achieved (20). From the time a fungal infection was suspected, our patient had uninterrupted treatment with VCZ according to the recommended dosing schedule in the drug's medical specialist information sheet. Apart from 3 days in the period from day +16 until day +69, he was treated with the intravenous solution of VCZ with a daily dose of 8-12 mg/kg body weight. Surprisingly, trough levels >2 mg/L were measured only once, perhaps indicating a rapid metabolization of VCZ in this patient. In the sample obtained from the cardiac thrombus, the minimal inhibitory concentration (MIC) of the *Fusarium* species was >8 mg/L for VCZ and also for isavuconazole, as determined by the German Reference Center for Invasive Mycotic Infections. This MIC was far beyond the generally accepted VCZ trough concentration of 2–5 mg/L. Posaconazole had previously been found resistant in this patient.

Allogeneic HSCT in patients with VSAA is usually associated with a favorable outcome of 80% overall survival, particularly when using bone marrow from an HLA-identical sibling (41). Several factors may have contributed to the death of the patient reported here. We assume that the critical events were the long period of neutropenia before his allogeneic HSCT in combination with the colonization by *Fusarium* species in the rural area where he grew up. From the time of diagnosis, he regularly needed packed red cell and platelet transfusions. Although the patient noticed these symptoms and therapeutic measures due to his bone marrow failure syndrome, the elevated risk for infections was not quite perceivable and delayed the decision to proceed to bone marrow transplantation.

In this case report, many questions remain unsolved and limit the reliable assessment of all events. First, it was not possible to define the time of colonization with the *Fusarium* species. Pre-transplant microbiological tests included routine screening for multi-resistant bacteria and swabs from the throat, but not swabs from other regions of the body. We think that colonization took place in the domestic area with close contact to animals and grain. A source inside the hospital appears unlikely, as there have been no further infections by *Fusarium* species in the past 10 years. Second, although splenic rupture was a major event and has not been reported before in patients with fusariosis, the most critical condition was the development of fungal endocarditis with subsequent embolism into the brain. Neither persistent treatment with VCZ nor the presence of neutrophils was able to prevent these complications. Third, it remains a matter of discussion whether higher doses of VCZ, a switch to isavuconazole or a transesophageal echocardiography at an earlier date would have resulted in a different outcome.

In conclusion, despite the uninterrupted administration of antimycotic substances, disseminated fusariosis remains a challenging and life-threatening condition in immunocompromised patients after allogeneic HSCT. Uncommon manifestations of this disease need to be considered at any time during the transplant procedure. Affected patients should undergo thorough examinations, including CT scans, MRI of the brain, ultrasound, funduscopy, and particularly echocardiography, which allows a reliable assessment of the heart valves.

## Data Availability

The original contributions presented in the study are included in the article/Supplementary Material, further inquiries can be directed to the corresponding author/s.
